# Video capsule endoscopy in inflammatory bowel disease

**DOI:** 10.1002/deo2.26

**Published:** 2021-08-16

**Authors:** Kenji J.L. Limpias Kamiya, Naoki Hosoe, Yukie Hayashi, Takaaki Kawaguchi, Kaoru Takabayashi, Haruhiko Ogata, Takanori Kanai

**Affiliations:** ^1^ Division of Gastroenterology and Hepatology Department of Internal Medicine Keio University School of Medicine Tokyo Japan; ^2^ Center for Diagnostic and Therapeutic Endoscopy Keio University School of Medicine Tokyo Japan

**Keywords:** capsule endoscopy, Crohn's disease, inflammatory bowel disease, pan‐enteric capsule endoscopy, ulcerative colitis

## Abstract

Since its introduction into clinical practice in 2000, capsule endoscopy (CE) has become an important procedure for many pathologies of small bowel (SB) diseases, including inflammatory bowel disease (IBD). Currently, the most commonly used capsule procedures are small bowel capsule endoscopy (SBCE), colon CE (CCE), and the recently developed pan‐enteric CE that evaluates the SB and colon in patients with Crohn's disease (CD). SBCE has a higher diagnostic performance compared to other radiological and conventional endoscopic modalities in patients with suspected CD. Additionally, CE plays an important role in monitoring the activity of CD in SB. It can also be used in evaluating response to anti‐inflammatory treatment and detecting recurrence in postsurgical patients with CD who underwent bowel resection. Due to its increasing use, different scoring systems have been developed specifically for IBD. The main target with CCE is ulcerative colitis (UC). The second‐generation colon capsule has shown high performance for the assessment of inflammation in patients with UC. CCE allows noninvasive evaluation of mucosal inflammation with a reduced volume of preparation for patients with UC.

## INTRODUCTION

Until the introduction of the first wireless capsule endoscopy (CE) method, called M2A (i.e., “mouth to anus”), in 2000 by Iddan et al.,^1^ the small bowel (SB) was considered the “black box” of the gastrointestinal (GI) tract because the SB was unattainable and only short‐segment imaging was possible. CE has revolutionized SB imaging as it is a noninvasive and patient‐friendly procedure. Currently, the indication for CE is mainly the investigation of obscure GI bleeding, identification of SB malignant tumors, and follow‐up of intestinal polyposis syndromes. It has increasingly become a tool for the evaluation of inflammatory bowel diseases (IBDs), mainly Crohn's disease (CD).

This review summarizes the role of CE in the diagnosis and assessment of IBD.

## CE DEVICES

Several CE platforms are currently available globally. The original and first CE, the PillCam, was manufactured by Given Imaging Ltd. (Yokne'am Illit, Israel). After its creation, several CE systems were created by other manufacturers, including the MiroCam (Seoul, Korea), EndoCapsule (Olympus, Tokyo, Japan), Omon (Jianshan Science & Technology, Chongqing, China), and CapsoCam (Capsovision, Saratoga, CA, USA).

After the introduction of the original SBCE procedure, there have been improvements in the system, such as higher resolution images, longer battery life, wider angle of view, adaptive frame rate, and the opportunity to perform an analysis in real time to increase the diagnostic yield.

The first colon CE (CCE) procedure was introduced in 2006 by Eliakim et al.^2^ as capable of visualizing the colon in a noninvasive way. The first‐generation CCE (CCE‐1; PillCam Colon, Given Imaging) had moderate sensitivity when detecting polyps ≥6 mm. For this reason, the second‐generation CCE (CCE‐2) was developed^3^ with a new technology, increasing the capsule frame rate from 4 to 35 images/s, allowing adequate imaging of the mucosa when the capsule is accelerated by peristalsis.

Recently, the new PillCam Crohn's capsule (Medtronic, Dublin, Ireland) was created to supply information on pan‐enteric disease distribution and the burden of the SB and colon.^4^

The patency capsule (PC), a dissolvable capsule of the same size as the PillCam SB, was developed for use as a prescreening tool to reduce the risk of capsule retention. There are two types of PC: a PC equipped with a radio‐frequency identification (RFID) tag to be identified with a scanner and the PC Japanese version, which does not have RFID to avoid the impact of the RFID tag in the stenosis.^5^ The effectiveness of PC use has been reported.^5^, ^6^, ^7^, ^8^, ^9^, ^10^

## CE IN CD

CD is a chronic inflammatory disease that can affect the entire GI tract but most commonly affects the SB.^11^ Between 70% and 90% of patients with CD also have involvement with SB,^12^ and 30% have an exclusive form of SB disease.^13^ Particularly, jejunal disease is considered a risk factor for strictures and is associated with a large number of surgical procedures.^14^ For this reason, the evaluation of SB has become of great interest for the diagnosis and management of CD.

In the past, evaluating the SB was a very difficult task because of the lack of visualization of the mucosa by conventional methods; however, since the introduction of the CE in 2000,^1^ better evaluation of the SB has been possible.

Generally, CE, magnetic resonance enterography (MRE), computed tomography enterography (CTE), and device‐assisted enteroscopy (DAE) are the preferred methods for the evaluation of SB.

### CE in suspected CD

Typically, CD is diagnosed according to clinical symptoms and using a combination of endoscopic, histological, radiological, and biochemical studies.^15^ Usually, ileocolonoscopy (IC) with biopsies and imaging studies are recommended as the gold standard to confirm the diagnosis; however, 30% of patients will have CD located beyond the reach of the ileocolonoscope, and it is in this group that CE may be useful.^13^

Mucosal features that may be seen with CE for CD include erythema, aphthous ulceration, loss of villi, villous edema, and longitudinal ulcers and strictures.^16^ These findings, however, are not specific for CD and may be seen in other types of SB enteropathy. Other enteropathies with similar mucosal CD appearance include SB lymphoma, intestinal tuberculosis, Behcet's disease, and enteropathy associated with human immunodeficiency virus and opportunistic infections.^17^ The International Conference on Capsule Endoscopy suggested that patients with suspected CD should have clinical diagnostic criteria, which are chronic diarrhea, weight loss, abdominal pain, evidence of elevated inflammatory biomarkers, or abnormal imaging studies suggestive of CD.^18^

Recently, published guidelines recommended SBCE for patients with clinical features consistent with CD and a negative IC, negative imaging studies, and absence of obstructive symptoms.^19^, ^20^, ^21^, ^22^ The European Society of Gastrointestinal Endoscopy (ESGE) suggests careful patient selection based on monitoring of symptoms and fecal/serologic biomarkers to improve the level of accuracy and yield of CE in patients with suspected CD.^21^

A meta‐analysis of 19 trials revealed that SBCE has a significantly increased diagnostic yield compared with IC (22%; 95% confidence interval [CI], 5–39%; *p* < 0.00001), radiography (32%; 95% CI, 16–48%; *p* < 0.00001), and CTE (47%; 95% CI, 31–63%; *p* = 0.009), but not with MRE (10%; 95% CI, 14–34%; *p* = 0.43) in nonstricturing SBCD^23^; however, other studies reported that CE was significantly superior to MRE for detecting SB lesions, especially in patients without endoscopic or clinical suspicion of stenosis.^24^, ^25^ SBCE has been incorporated into several guidelines, consensus proceedings, and recommendations as a first‐line method for suspected isolated SBCD.^19^, ^20^, ^21^, ^26^, ^27^

One of the main concerns for physicians is the retention of the CE in the GI tract. Currently, in Japan, the agile tag‐less PC is used.^5^ Patients with symptoms suggestive of SB obstruction, a known narrowing of the intestinal lumen or strictures, benefit the most from the PC test. Currently, there are two different criteria for PC administration in patients with CD: a selective approach administered only to patients with obstructive symptoms and a nonselective approach administered to all patients with CD. Nemeth et al.^28^ reported a 1.5% retention risk in SB without previous use of PC and 2.1% after a negative examination of PC (*p* = 0.9). They also reported that the risk of CE retention was not reduced by nonselective PC use in asymptomatic patients, and a positive PC study was significantly associated with CE retention.

Most cases of CE retention are asymptomatic. In these cases, a trial is commonly conducted by administering steroids to reduce inflammation and allow expulsion of the CE capsule. If this test is unsuccessful, the CE capsules are normally removed by DAE. Approximately 32–45% of cases will need surgery.^29^, ^30^

### CE in known CD

In most patients, the CD phenotype changes from diagnosis over time that progress from inflammatory lesions to structuring or penetrating disease.^31^ During the last years, the treatment objective for CD has been changing from having clinical control of symptoms to having the inflammation reversed and achieving mucosa healing.^32^ CE has the advantage of being able to identify the presence of an active CD that would not be evident from conventional markers or radiological images. Ben‐Horin et al. found that CE follow‐up can predict flares within 6 months in patients without symptoms.^33^

C‐reactive protein (CRP) and fecal calprotectin (FC) are markers of inflammation frequently used to monitor IBD activity. Studies have evaluated the correlation between inflammation biomarkers and CE findings. Kopylov et al. demonstrated in a study that only 15.4% of patients achieved mucosal healing in the SB. In addition, CRP and FC had a low correlation with active SB inflammation.^34^ Another study involving 43 patients with symptomatic CD determined by clinical indices, CRP, FC, and CE Crohn's Disease Activity Index (CECDAI) score at the beginning and 52 weeks of treatment showed that the biochemical response was correlated with endoscopic remission in only 42% of patients; the results were confirmed in another study.^35^, ^36^

Studies on patients with CD who underwent a CE procedure with symptoms such as unexplained anemia, malnutrition, or inconsistency between symptoms and IC suffered changes in therapeutic management due to lesions found only with CE.^37^, ^38^, ^39^ Min et al. reported that 75–86% of pediatric patients with abnormalities in CE findings had treatment intensified by adding an anti‐TNF agent, which demonstrated clinical improvement and a better biological status after 1 year.^40^ This was also demonstrated by Oliva et al. in a prospective study conducted in Italy.^41^

Based on these studies, we believe that CE could be a useful tool in the therapeutic management of patients with CD.

Pan‐enteric surveillance for CD has been reported.^42^ Several reports evaluated the performance of the CCE‐2 and SB colon (SBC) capsule (PillCam Crohn's capsule; Medtronic) in a pan‐enteric examination in CD (Table 1).^43^, ^44^, ^45^, ^46^ D'Haens et al. compared the CCE‐2 with colonoscopy (CS) in 40 patients with active CD. The study demonstrated a good correlation in assessing the CD endoscopic index of severity (intraclass correlation coefficient, 0.65; 95% CI, 0.43–0.80) and high sensitivity (86%) but low specificity (40%).^43^ Another study that compared the CCE‐2 with IC, MRE, and SB ultrasonography in pediatric patients demonstrated that the CCE‐2 was superior for the detection of colonic lesions, with a sensitivity, specificity, and a positive and negative predictive value of 89, 100, and 100 and 91%, respectively.^45^

**TABLE 1:** Studies compared small bowel colon (SBC) capsule with MRE and/or ileocolonoscopy in confirmed CD patients

**First author**	**Capsule generation**	**Sample size**	**Disease Status**	**Compared modality**	**Activity score standard**	**Diagnostic value**
Bruining^49^	PCC	119	Known CD	MRE and/or IC	SB: LS	Se/Sp/PPV/NPV (%)
					TI and colon: SES‐CD	PCC:94/74/91/83
						MRE and/or IC: 100/22/77/100
Leighton^47^	PCC	66	Known CD	IC	N/R	Diagnostic yield
						83.3% versus 69.7%
Tjandra^44^	CCE‐2	34	Known CD	IC	SES‐CD	Correlation rate: 0.599
D'Haens^43^	CCE‐2	40	Known CD	IC	CDEI‐S	Correlation rate:
					SES‐CD	CDEI‐S: ICC = 0.65
					GELS	SES‐CD: ICC = 0.50
						GELS: ICC = 0.40
						Se/Sp: 86%/40%
Oliva ^45^	CCE‐2	40 (pediatric)	Known CD	MRE and/or IC	SB: LS	Se/Sp/PPV/NPV (%)
					Colon: SES‐CD	Colon: 89/100/100/91
						SB: 90/94/95/90
						Total: 89/92/96/79
Hall ^46^	CCE‐2	10	Known CD	IC	SB: CECDAI	Correlation rate:
					Colon: SES‐CD	SB: (CCE + SBCE) = 0.896 Colon: (CCE + colonoscopy) = 0.667

**Abbreviations**: CCE, colon capsule endoscopy; CCE‐2, second‐generation colon capsule endoscopy; CD, Crohn's disease; CDEI‐S, Crohn's disease endoscopic index of severity; CECDAI, capsule endoscopy Crohn's disease activity index; GELS, global evaluation of lesion severity; IC, ileocolonoscopy; ICC, intra‐class correlation coefficient; IL, ileocolonoscopy; LS, Lewis score; MRE, magnetic resonance enterography; NPV, negative predictive value; N/R, not reported; PCC, Pillcam Crohn's capsule; PPV, positive predictive value; SB, small bowel; SBCE, small bowel capsule endoscopy; Se, sensitivity; SES‐CD, simple endoscopic score for Crohn's disease; Sp, specificity; TI, terminal ileum.

The SBC capsule (PillCam Crohn's capsule; Medtronic) was recently designed to assess the SB and colon.^47^ In a multicenter study, Tai et al. concluded that the use of the SBC capsule was feasible in routine practice, and its ability to detect proximal SB disease may allow a better estimate of prognosis and intensification of treatment.^48^ Leighton et al.^47^ compared the diagnostic yield of SBC capsules with IC in patients with active CD. The diagnostic yield for active CD lesions was 83.3% for the SBC capsule and 69.7% for IC (yield difference, 13.6%; 95% CI, 2.6–24.7). They concluded that the diagnostic yield for the SBC capsule may be higher than IC. Bruining et al., in a recent multicenter prospective study, also reported the utility of the SBC capsule in comparison with IC and/or MRE. The sensitivity of the SBC capsule was higher than MRE for proximal SB inflammation (97% vs. 71%, *p* = 0.021) and similar to MRE and/or IC in the terminal ileum and colon (*p* = 0.500–0.625).^49^ At present, the usefulness of the CCE‐2 and SBC capsules is uncertain. Large‐scale studies are needed.

Asymptomatic recurrence of CD after ileal resection can occur in up to 70% of cases within a year after surgery.^50^ De Cruz et al. demonstrated that early detection of endoscopic recurrence is the key to starting biological therapy, preventing clinical recurrence, and avoiding a new surgery.^51^ The standard method for surveillance in postsurgical patients is IC; however, studies have shown that CE can detect recurrence in places unreachable with IC.^52^ In their study, Pons Beltran et al. demonstrated that CE detected recurrence in 62% of patients compared with only 25% in patients undergoing IC.^53^ Similar results were found by Sorrentino et al., and this understanding led to improvement in management in 52% (12/23) of patients. Shiga et al., in a recent study, found less risk of hospitalization, repeat surgery, or need for endoscopic dilation in the group of patients who underwent CE as a follow‐up after surgery compared with patients who did not have CE follow‐up.^54^ For this reason, monitoring lesions with CE that are beyond the scope could be more beneficial because it is a noninvasive method for the postsurgical patient.

### CE scoring systems in CD

Currently, there are two validated indexes available to quantify the burden of SB inflammation: the CECDAI and the Lewis score (LS). CECDAI, proposed by Gal et al. in 2008 (Table 2), consists of dividing the SB into proximal and distal segments according to the transit time of the SB. Each segment is assigned an inflammation score (A, 0–5), an extension score (B, 0–3), and a stenosis score (C, 0–3), which are then totaled as A × B + C. The total score, ranging from 0 to 36, is the sum of two segments. The authors, however, did not establish a specific threshold for the definition of mucosal inflammation, although a higher CECDAI is considered to represent more severe mucosal inflammation.^55^

**TABLE 2:** Capsule endoscopy Crohn's disease activity index^55^

**A: Inflammation**	**B: Extent**	**C: Stricturing**
0 = None	0 = None	0 = None
1 = Mild to moderate/edema/hyperaemia/denudation	1 = Focal	1 = Single (passed)
2 = Severe edema/hyperaemia/denudation	2 = Patchy	2 = Multiple (passed)
3 = Small ulcer (5 mm)	3 = Diffuse	3 = Obstructing
4 = Moderate ulcer (5–20 mm)		
5 = Large ulcer (20 mm)		

Score for each segment: A x B + C

LS, first reported by Gralnek et al. in 2008 (Table 3),^56^ is currently embedded in the software of the Medtronic capsule. It scores inflammatory changes in the mucosa of the SB using three parameters: villous edema, ulceration, and stenosis. Villous edema is considered to exist when the broad dimension of the villi is equal to or greater than the vertical or horizontal dimension. Ulceration is identified as a break in the mucosa with a red, pinkish‐white, or yellow ulcer base. In SBCE, the SB transit time is divided into three quantiles. In each quantile, the number, extent, and size of villus edema and ulceration are multiplied, and then the largest quantile value is added to a separate stenosis score. To quantify the severity of mucosal changes, <135 is considered normal or clinically insignificant inflammation, ≥135 to <790 is considered mild inflammation, and ≥790 is considered moderate to severe inflammation. Yablecovitch et al. compared LS and CECDAI scores and found a strong correlation between both scores but a moderate correlation with FC.^57^ Omori et al., in a recent study, compared the two scores and concluded that the values of LS 135 and 790 were equivalent to the values of 4.9 and 6.9 with CECDAI scores. There was a strong correlation between the two scores; however, the CECDAI score was more reflective of high clinical activity and extensive inflammation.^58^

**TABLE 3:** Lewis capsule endoscopic scoring index^56^

	**Parameters**	**Number**	**Longitudinal extent**	**Descriptors**
First tertile	Villous appearance	Normal = 0	Short segment = 8	Single = 1
		Edematous = 1	Long segment = 12	Patchy = 14
			Whole segment = 20	Diffuse = 17
	Ulcer	None = 0	Short segment = 8	<1/4 = 9
		Single = 3	Long segment = 12	1/4 to 1/2 = 12
		Few = 5	Whole segment = 20	>1/2 = 18
		Multiple = 10		
Second tertile	Villous appearance	Normal = 0	Short segment = 8	Single = 1
		Edematous = 1	Long segment = 12	Patchy = 14
			Whole segment = 20	Diffuse = 17
	Ulcer	None = 0	Short segment = 8	<1/4 = 9
		Single = 3	Long segment = 12	1/4 to 1/2 = 12
		Few = 5	Whole segment = 20	>1/2 = 18
		Multiple = 10		
Third tertile	Villous appearance	Normal = 0	Short segment = 8	Single = 1
		Edematous = 1	Long segment = 12	Patchy = 14
			Whole segment = 20	Diffuse = 17
	Ulcer	None = 0	Short segment = 8	<1/4 = 9
		Single = 3	Long segment = 12	1/4 to 1/2 = 12
		Few = 5	Whole segment = 20	>1/2 = 18
		Multiple = 10		
Stenosis		None = 0	Non‐ulcerated = 2	Traversed = 7
		Single = 14	Ulcerated = 24	Non‐traversed = 10
		Multiple = 12		

Few: Two to seven lesions; Long segment: 11–50% of a tertile; Multiple: Eight or more ulcers, two or more stenoses; Short segment: ≤10% of the tertile; Whole tertile: ≥50% of the tertile.

Recently, Eliakim et al.^59^ proposed a new quantitative scoring system using the PillCam Crohn's capsule to monitor pan‐enteric mucosal inflammation in CD; it was called the PillCam Crohn's Score (PCCS). The authors concluded that the PCCS correlated well with LS (0.9; *p* < 0.0001), had good reliability, and could potentially be more accurate in estimating the pan‐enteric inflammatory burden. More studies are necessary to evaluate the real performance of this new pan‐enteric score.

Currently, there is no validated or accepted standard CE score to be used in practice.

### CE in UC

Ulcerative colitis (UC) is an idiopathic chronic IBD with remission and relapse episodes associated with impaired quality of life.^60^ Endoscopically confirmed mucosal healing has resulted in a favorable prognosis, long clinical remission, fewer bowel resection surgeries, and steroid‐free clinical remission.^13^ Conventional CS is the gold standard in the diagnosis and evaluation of the severity and extent of the disease and for the dysplasia surveillance in patients with UC.^61^, ^62^, ^63^ However, CS is an invasive and uncomfortable procedure for the patient requiring sedation. Vienne et al. reported that only 54% of patients with IBD received CS in an observational period of 4 years.^64^

The CCE‐1 has not been as expected when compared with conventional CS. The CCE‐2 was improved in terms of image quality and provides a wide viewing angle compared with the CCE‐1. Hosoe et al. showed a good correlation between CCE‐2 and conventional CS.^65^ Several studies have evaluated the performance of CCE for the evaluation of the severity of inflammation in patients with UC.^66^, ^67^, ^68^

Hosoe et al. conducted the first feasibility study of CCE‐2 for the evaluation of the severity of mucosal inflammation in patients with UC. They found that the CCE‐2 procedure completed within 8 h occurred in 69% of patients, and a good to excellent level of colon cleansing using a low‐volume polyethylene glycol (PEG) solution and prokinetic agents was experienced by less than 50% of patients. They found a significant correlation (*p* = 0.797) between the CCE‐2 images determined by the Matts endoscopic score and CS. One of the reasons the authors associated the existing significant correlation is because inflammation in UC was not patchy, but rather diffusive, which could be detectable in CCE‐2 images. They believe that a high level of colon cleansing is not necessary when evaluating inflammatory severity in UC.^69^

Oliva et al.^70^ reported a higher diagnostic accuracy of the CCE‐2 during the evaluation of disease activity compared with CS in pediatric patients, also confirmed by Shi et al. in a large‐scale prospective study.^71^

Interestingly, Hisabe et al.^72^ reported that 36% of lesions of a patient with UC were detected in the SB, as well as 27.8% of patients also reported by Ninomiya et al.^73^ We believe that the CE is also a useful device to identify injuries that could occur in the SB in patients with UC.

Currently, the CCE‐2 has great potential to become an inflammation monitoring tool in UC. However, this tool would not be appropriate for the detection of colitis associated with cancer. We believe that more studies are necessary to demonstrate the usefulness of CE in patients with UC.

### CCE scoring systems in UC

The Ulcerative Colitis Endoscopic Index of Severity,^74^ Mayo Endoscope Scores,^75^ and other scoring systems are used to evaluate the severity of UC in clinical practice.

Hosoe et al. developed a Capsule Scoring of Ulcerative Colitis (CSUC).^76^ Although not yet validated, it is currently the only CCE score used to evaluate inflammation in UC. The score consists of dividing the colon into the proximal and distal regions. The proximal region ends in the splenic flexure. The items to be evaluated are “vascular pattern,” “bleeding,” and “erosions and ulcers.” The final score, called CSUC, is calculated using the sum “vascular pattern total (proximal + distal) + total bleeding + erosions and ulcers in total” (lower value–higher value, 0–14). Matsubayashi et al. evaluated the usefulness of the CSUC score in predicting the relapse of inactive UC. Patients who relapsed within 1 year had a CSUC value that was higher compared with patients who maintained clinical remission (2.83 ± 1.95 vs. 0.72 ± 1.00; *p* < 0.01). They concluded that CSUC is useful in predicting future relapses within 1 year in patients with UC who are in clinical remission.^77^

Currently, although guidelines do not support the use of CE in patients with UC, and it is unlikely that it will replace fecal biomarkers or IC as the first line of investigation for UC, it is still considered a viable alternative.

More clinical studies are needed to evaluate the performance of CE in UC.

### Bowel preparation of CCE for UC

One of the aspects of less acceptance at the time of performing CCE in patients with UC is the large volume of laxatives that the patient needs to ingest.^78^ The standard volume of preparation for CCE colon polyp surveillance consisted of 4–6 L of laxatives.^79^, ^80^, ^81^ Different studies were conducted with reduced bowel preparation regimens to increase the completion rate and improve the colon cleansing efficacy and patient acceptance. A PEG solution containing ascorbic acid (PEG‐ASC) was recently used as a reduced preparation for UC.^82^, ^83^ Okabayashi et al. proposed that a simple 1‐day reduced regimen for the CCE‐2 would be more accepted by patients. It consisted of a maximum total of 3 L of fluid (PEG‐ASC, 2 L; water, 1L), achieving a total observation rate of 94%.^83^

## CONCLUSION

CE has become an important complementary noninvasive, well‐tolerated tool that is not only used for making an early diagnosis but also to provide a good prognosis stratification. CE will help optimize treatment strategies in patients with CD.

The great limitation for CE in UC is the large volume of intestinal preparations required, reducing the acceptability of patients, and increasing the impossibility of taking biopsies.

A common problem is the time spent reading and interpreting injuries and the high possibility of overlooking injuries. Currently, the development of the automatic diagnosis of CE using artificial intelligence is under evaluation.^84^, ^85^, ^86^ Technology that allows obtaining biopsies or possibly drug delivery is necessary and ideal for those patients with IBD who permanently need to undergo endoscopy.

## CONFLICT OF INTEREST

Author N.H. is an associate editor of *DEN Open*. Other authors declare that they have no conflict of interest.
